# Splenic Injury Following Routine Colonoscopy: A Case Report

**DOI:** 10.7759/cureus.107295

**Published:** 2026-04-18

**Authors:** Mallory T Bruskas, Mohamed Elfedaly, Matthew Ellis, Andrea Aldaz Payan

**Affiliations:** 1 Department of Surgery, Texas Tech University Health Sciences Center, Amarillo, USA

**Keywords:** colonoscopy complication, screening colonoscopy, splenic angioembolization, splenic laceration, surgical acute abdomen

## Abstract

Splenic injury is a rare but potentially life-threatening complication of colonoscopy procedures. We present the case of a 64-year-old female patient who developed an acute splenic laceration following an elective screening colonoscopy. The procedure was anatomically challenging due to colonic tortuosity but otherwise uneventful. While in the post-anesthesia care unit, the patient became hypotensive with abdominal distention and tenderness. Initial abdominal radiography was unrevealing for acute abnormalities. However, CT with intravenous contrast demonstrated hemoperitoneum secondary to a small inferior pole splenic laceration with active arterial extravasation. The patient was resuscitated with two units of packed red blood cells and treated with interventional radiology-guided angioembolization. The patient was then monitored in the surgical intensive care unit (SICU), experienced no further bleeding, and was able to be discharged home on hospital day three without further complications. This case highlights the importance of maintaining a high index of suspicion for splenic injury in patients presenting with procedures complicated by colonic tortuosity or with postoperative unexplained abdominal pain or hemodynamic instability following colonoscopy. Early recognition and prompt intervention, combined with minimally invasive management, can prevent the need for more invasive procedures, such as splenectomy, and result in favorable outcomes.

## Introduction

Routine screening colonoscopies remain the standard of care in screening for colon cancer in all individuals beginning at age 45 until age 75, in individuals who have an average lifetime risk for developing colon cancer [1]. Routine screening colonoscopy carries a low but measurable risk of serious complications, including approximately 3.5 to 7.3 perforations per 10,000 colonoscopies performed and 6.5 to 23.1 major bleeding events per 10,000 colonoscopies performed in the average-risk population [2].

Splenic laceration after routine colonoscopy is extremely rare, occurring at a rate of 0.20 to 0.34 per 10,000 procedures [2]. A recent large registry study of outpatient colonoscopies found an incidence of 3.19 splenic injuries per 100,000 colonoscopies, approximately one in 31,400 patients [3]. Despite its rarity, this complication carries significant morbidity and a 30-day mortality rate of 36.1 per 1,000 splenic injuries [2].

We present a rare case of splenic injury after a routine screening colonoscopy in a 64-year-old female patient. The patient developed hypotension, abdominal pain, and distention due to a small inferior pole splenic laceration after a routine colonoscopy. She was successfully managed with angioembolization and close observation in the surgical intensive care unit (SICU). 

This case underscores the critical importance of early recognition and timely management of acute splenic injury, a potentially life-threatening complication of colonoscopy. Given the nonspecific initial presentation and the increasing utilization of colonoscopy for both screening and diagnostic purposes, heightened clinical awareness is essential to prevent delays in diagnosis and reduce morbidity. This report contributes to the limited existing literature by reinforcing key diagnostic considerations and management strategies while emphasizing the need for vigilance among clinicians.

## Case presentation

**Figure 1:**
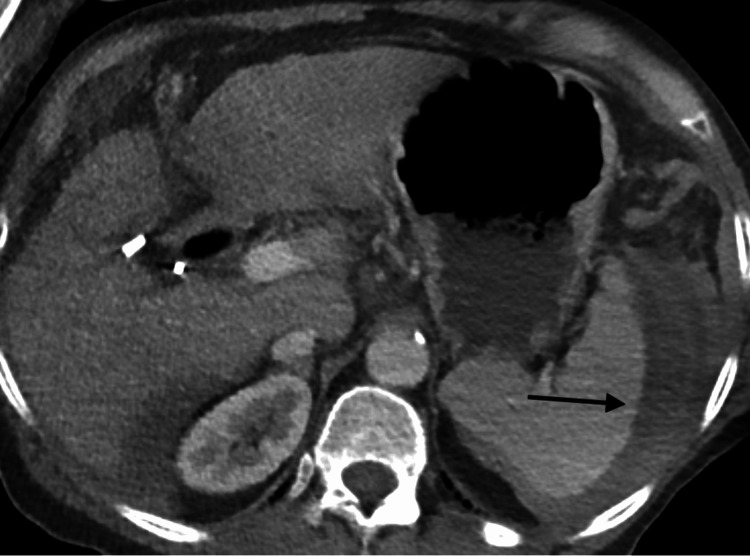
CT scan with IV contrast of the abdomen and pelvis demonstrating a splenic laceration The black arrow is pointing towards the area of hemorrhage from an inferior pole splenic laceration. The fluid about the spleen is hyperdense, and additionally, there is a focus of contrast just inferior to the inferior pole with associated hemoperitoneum.

A 64-year-old female patient with a history of liver cirrhosis presented to the hospital for an elective screening colonoscopy. She had no history of prior abdominal surgeries, and the only medication use was spironolactone for her cirrhosis, which was diagnosed six years prior. The procedure was challenging due to colonic tortuosity, requiring extensive maneuvers, torquing, withdrawing, and advancing multiple times for successful completion of the procedure. Two hours after completion of the colonoscopy, the patient was noted to be hypotensive with a distended and tender abdomen in the post-anesthesia care unit (Table 1). An abdominal X-ray was obtained, demonstrating no free air under the diaphragm. However, due to a high clinical suspicion for an underlying injury, a CT scan with intravenous contrast was performed, revealing a hemoperitoneum secondary to a small inferior pole splenic laceration with active arterial extravasation (Figure 1).

**Table 1:** Vital signs following colonoscopy SpO_2_: peripheral oxygen saturation

Vital Sign	Measurement	Reference
Blood Pressure (Systolic/Diastolic)	82/54 mmHg	90/60 mmHg to 120/80 mmHg
Temperature	98.2 F	97.8 F to 99 F
Respiratory Rate	25 breaths per minute	12 to 20 breaths per minute
SpO_2_	95% on a two-liter nasal cannula	95%-100% on room air

**Figure 2:**
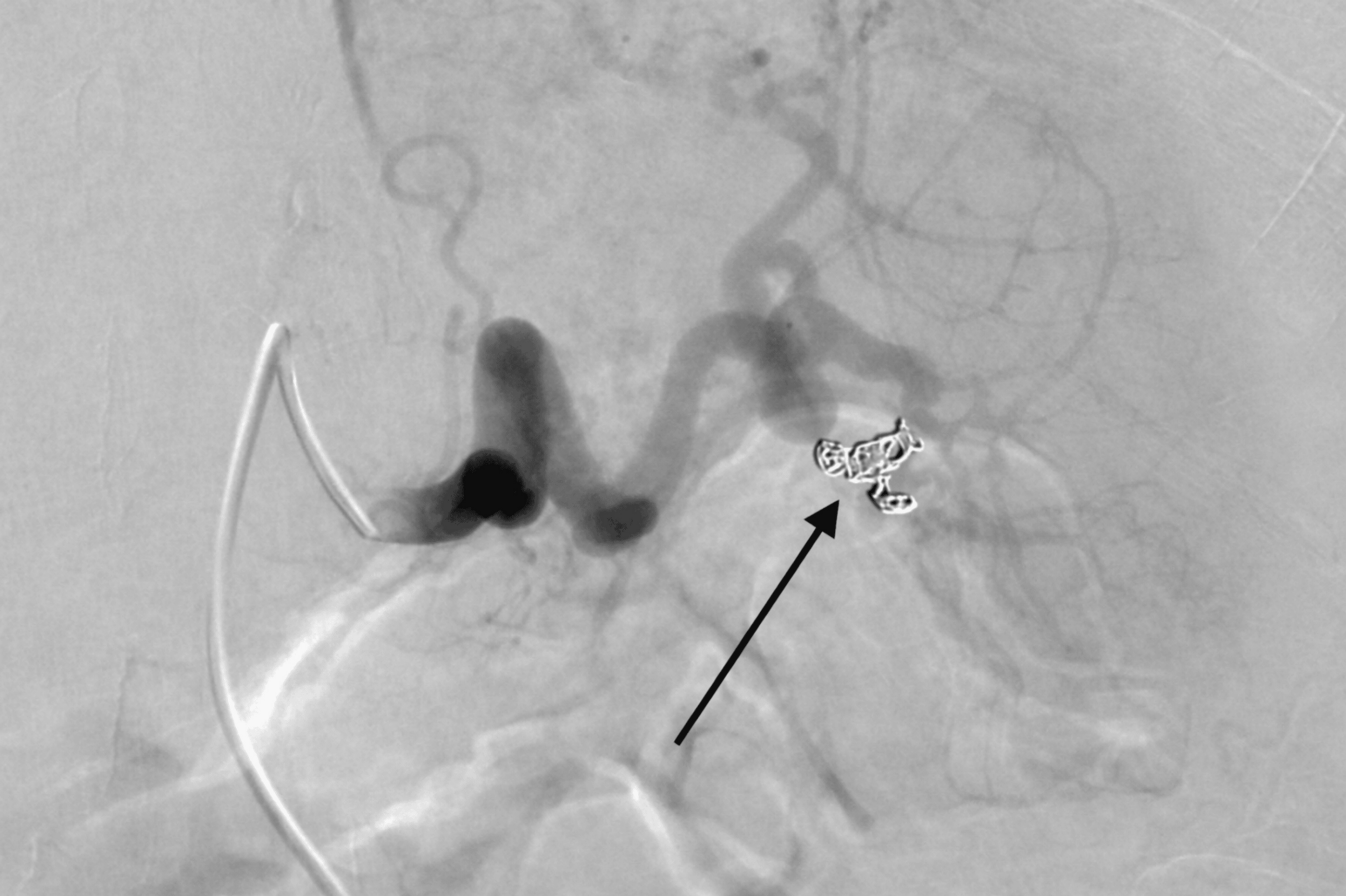
Angiography post angioembolization Splenic angiogram performed after the microcatheter was withdrawn with the black arrow demonstrating complete occlusion of the splenic artery branch vessels supplying the inferior splenic pole. No active bleeding appreciable.

A complete blood count (CBC) was obtained, which demonstrated mild anemia and thrombocytopenia but no evidence of acute infection (Table 2). Following the diagnosis of acute splenic laceration, the patient promptly received two units of packed red blood cells (PRBCs) and underwent emergent selective angioembolization of the inferior pole artery of the spleen. Angiography performed post embolization showed successful cessation of active bleeding (Figure 2).

**Table 2:** Complete clood count performed following colonoscopy WBC: white blood cell; RBC: red blood cell; MCV: mean corpuscular volume

Lab	Value	Reference Range
WBC (×10³/µL)	6.7	4.0-10.0
Hemoglobin (gm/dL)	10.9	12.1-15.1
RBC (×10⁶/µL)	3.38	4.2-5.4
Hematocrit (%)	33	36-48
MCV (fL)	98	80-100
Platelet (×10³/µL)	91	150-450

Following angioembolization, the patient was admitted to the SICU for postoperative monitoring. After 24 hours, her hemoglobin levels remained stable, and her blood pressure normalized (Tables 3-4). Labs were also significant for post-transfusion platelet count of 60 × 10^3^/mcL, but on further investigation, this was found to be slightly below the patient's baseline, likely secondary to her chronic liver cirrhosis (Table 5). 

**Table 3:** Vital signs 24 hours post angioembolization SpO_2_: peripheral oxygen saturation

Vital Sign	Measurement	Reference
Blood Pressure (Systolic/Diastolic)	103/68 mmHg	90/60 mmHg to 120/80 mmHg
Temperature	98.6 F	97.8 F to 99 F
Respiratory Rate	16 breaths per minute	12 to 20 breaths per minute
SpO_2_	95% on room air	95%-100% on room air

**Table 4:** Complete blood count performed 12 hours post angioembolization WBC: white blood cell; RBC: red blood cell; MCV: mean corpuscular volume

Lab	Value	Reference Range
WBC (×10³/µL)	5.8	4.0-10.0
Hemoglobin (gm/dL)	10.7	12.1-15.1
RBC (x10^6^/µL)	3.30	4.2-5.4
Hematocrit (%)	32	36-48
MCV (fL)	96	80-100
Platelet (x10^3^/µL)	60	150-450

**Table 5:** Complete blood count performed on routine labs one year prior to the colonoscopy WBC: white blood cell; RBC: red blood cell; MCV: mean corpuscular volume

Labs	Values	Reference Range
WBC (x10^3^/µL)	5.8	4.0-10.0
Hemoglobin (gm/dL)	14.6	12.1-15.1
RBC (x10^6^/µL)	4.46	4.2-5.4
Hematocrit (%)	45	36-48
MCV (fL)	101	80-100
Platelet (x10^3^/µL)	73	150-450

The patient was then subsequently transferred to the general surgical floor for continued observation. In the absence of further clinical concerns, her diet was gradually advanced to her regular diet, which she tolerated without issue. The patient was discharged on hospital day 3 following stable laboratory findings, stable vital signs, and no further reported complications (Tables 6-7).

**Table 6:** Vitals signs taken prior to discharge SpO_2_: peripheral oxygen saturation

Vital Sign	Measurement	Reference
Blood Pressure (Systolic/Diastolic)	100/60 mmHg	90/60 mmHg to 120/80 mmHg
Temperature	98.2 F	97.8 F to 99 F
Respiratory Rate	19 breaths per minute	12 to 20 breaths per minute
SpO_2_	100% on room air	95%-100% on room air

**Table 7:** Complete blood count obtained on postoperative day 3 WBC: white blood cell; RBC: red blood cell; MCV: mean corpuscular volume

Lab	Value	Reference Range
WBC (x10^3^/µL)	5.4	4.0-10.0
Hemoglobin (gm/dL)	10.9	12.1-15.1
RBC (x10^6^/µL)	3.24	4.2-5.4
Hematocrit (%)	30	36-48
MCV (fL)	93	80-100
Platelet (x10^3^/µL)	62	150-450

The patient was then seen in our outpatient clinic two weeks following hospital discharge, where she appeared well and reported no complaints. A repeat CBC was performed, which showed resolution of anemia and thrombocytopenia (Table 8). The decision was made not to perform repeat CT imaging due to the absence of clinical symptoms and a benign abdominal exam.

**Table 8:** Complete blood bount performed two weeks post hospital discharge WBC: white blood cell; RBC: red blood cell; MCV: mean corpuscular volume

Lab	Value	Reference Range
WBC (x10^3^/µL)	8.3	4.0-10.0
Hemoglobin (gm/dL)	14.2	12.1-15.1
RBC (x10^6^/µL)	4.34	4.2-5.4
Hematocrit (%)	40	36-48
MCV (fL)	93	80-100
Platelet (x10^3^/µL)	181	150-450

## Discussion

Important differential diagnoses to include in any patient experiencing post-colonoscopy hypotension and abdominal pain include colonic perforation, post-polypectomy hemorrhage, sedation-related hypotension, mesenteric ischemia, and splenic laceration [4]. Splenic injury after colonoscopy is an exceedingly rare but potentially life-threatening complication. While historical estimates suggested an incidence of 0.05% to 0.5%, more recent large-scale reviews and registry data indicate a lower rate, typically ranging from 0.0001% to 0.004% [5,6]. Female gender and previous abdominal surgeries, such as partial splenectomy, nephrectomy, and hysterectomy, are recognized as significant risk factors associated with a higher incidence of this injury [7]. While this patient had chronic liver cirrhosis, studies have demonstrated that this does not contribute to difficult anatomy during colonoscopies in patients without ascites, as was not found in this patient [8]. 

The primary mechanism of injury involves excessive traction on the splenocolic ligament or direct trauma during the navigation of the splenic flexure [9]. Technical aspects associated with increased risk include forceful or traumatic insertion of the colonoscope, excessive torque or twisting of the instrument, over-insufflation of air, performing polypectomy or biopsy in the proximal colon, and use of rigid or standard adult colonoscopes in patients with angulated splenic flexures [10].

Conversely, protective technical strategies include the use of variable stiffness colonoscopes and the avoidance of excessive force or traction during the procedure [11].

Management has shifted toward organ-preserving strategies with an emphasis on recognizing early signs of possible post-procedural hemorrhage to facilitate rapid diagnosis of complications via CT imaging [12]. Angioembolization is now an effective first-line management strategy for hemodynamically stable patients with active extravasation. Splenectomy is generally reserved for patients with hemodynamic instability refractory to resuscitation, cases of ongoing hemorrhage despite angioembolization, or when interventional radiology services are unavailable [13]. Current guidelines suggest against repeat imaging after splenic artery angioembolization for low-grade splenic injuries, recommending instead that decisions for repeat imaging be based on clinical findings due to low rates of management changes in asymptomatic patients [14].

## Conclusions

Although rare, splenic injury must remain a key diagnostic consideration in patients presenting with hypotension and abdominal pain following colonoscopy procedures, especially in the context of colonic tortuosity or difficult anatomy. This case highlights the vitality of early clinical recognition and prompt imaging to establish an accurate diagnosis. Timely intervention with angioembolization can achieve effective hemostasis while minimizing the need for more invasive procedures, such as splenectomy. Recognizing that no intervention is entirely benign encourages clinicians to remain vigilant during and after the procedure, ensuring that subtle post-procedural changes are not overlooked. A proactive, risk-aware approach is essential to safeguarding patient outcomes in all endoscopic practices.
